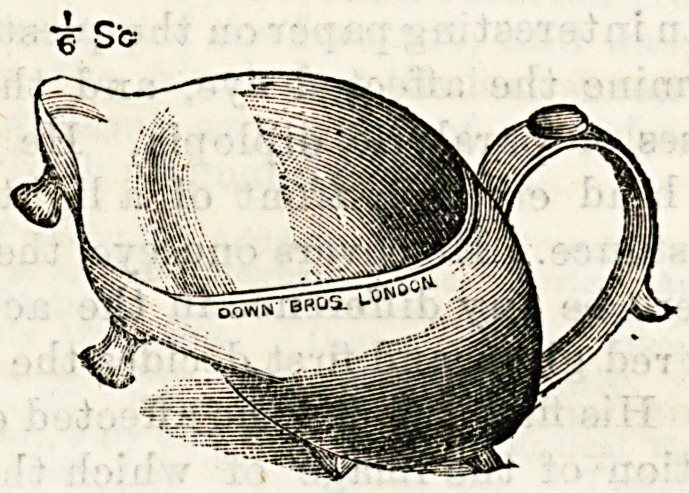# New Appliances and Things Medical

**Published:** 1895-02-02

**Authors:** 


### NEW APPLIANCES AND THINGS MEDICAL.

### A NEW DRESSING TRAY.

(Down Brothers, 5 and 7, St. Thomas's Street, S.E.)

The dressing tray before us supplies a distinct want. The usual shallow dressing tray in hospital use frequently proves inadequate and inconvenient where much irrigation is necessary, and we are not surprised that the inventor of the present article is a nurse, who must have practically felt the want of a more suitable utensil, especially in colotomy and empyema cases, for which it is particularly necessary. The illustration shows the special features of the tray. It can hold three pints of water, is so arranged as to rest steadily at different inclinations, is conveniently shaped to fit the curves of the body, whilst the prevention of splashing and the addition of a handle are obvious advantages. We feel sure that the tray will be considered indispensable when once it has been tried, and that it will be largely adopted for surgical use in hospitals and private nursing.

## Figures and Tables

**Figure f1:**
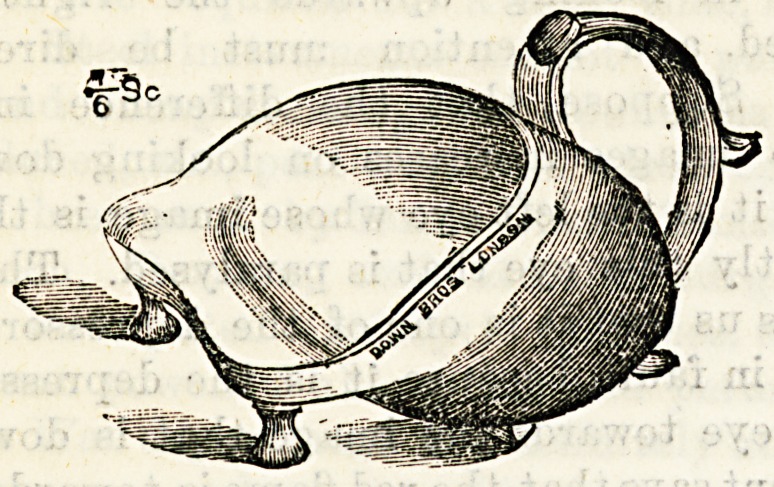


**Figure f2:**